# Within-class differences in cancer risk for sulfonylurea treatments in patients with type 2 diabetes (ZODIAC-55) – a study protocol

**DOI:** 10.1186/s12885-017-3433-z

**Published:** 2017-06-23

**Authors:** Dennis Schrijnders, Geertruida H. de Bock, Sebastiaan T. Houweling, Kornelis J. J. van Hateren, Klaas H. Groenier, Jeffrey A. Johnson, Henk J. G. Bilo, Nanne Kleefstra, Gijs W. D. Landman

**Affiliations:** 1 Diabetes Centre, Isala, P.O. Box 10400, 8000 GK Zwolle, the Netherlands; 2 Langerhans Medical Research Group, Zwolle, the Netherlands; 3 Department of Epidemiology, University of Groningen, University Medical Center Groningen, Groningen, the Netherlands; 4 Department of General Practice, University of Groningen, University Medical Center Groningen, Groningen, the Netherlands; 5 School of Public Health, University of Alberta, Edmonton, Canada; 6 Department of Internal Medicine, University of Groningen, University Medical Center Groningen, Groningen, the Netherlands; 7 Department of Internal Medicine, Isala, Zwolle, the Netherlands; 8 Department of Internal Medicine, Gelre Hospital, Apeldoorn, the Netherlands

**Keywords:** Type 2 diabetes, Sulfonylureas, Cancer, Within-class differences

## Abstract

**Background:**

Patients with type 2 diabetes (T2D) are at increased risk for developing cancer. As approximately 8% of the world’s population is living with T2D, even a slight increase in cancer risk could result in an enormous impact on the number of persons developing cancer. In addition, several glucose lowering drug classes for treating patients with T2D have been associated with a difference in risk of cancer overall, and especially for obesity related cancers. In what way and to what degree cancer risk is modified by the use of different sulfonylureas (SU) is unclear. The primary aim of this study will be to evaluate within-class SU differences in obesity related cancer risk. Secondary aims will be to investigate within-class SU differences in risk for all cancers combined and site-specific cancers separately (i.e. breast, colorectal, prostate, bladder and lung cancer) and to account for duration-response relationships between individual SU use and cancer risk.

**Methods:**

Patients will be selected from a Dutch primary care cohort of patients with T2D linked with the Dutch Cancer Registration (ZODIAC-NCR). Within this cohort study annually collected clinical data (e.g. blood pressure, weight, HbA1c) and nationwide data on cancer incidence are available. Time-dependent cox proportional hazard analyses will be performed to evaluate SU cancer risk, adjusted for potential confounders.

**Discussion:**

This study will be the first prospective cohort study investigating within-class SU differences in cancer risk and could contribute to improved decision making regarding the individual drugs within the class of SUs, and possibly improve quality of life and result in an increased cost-effectiveness of healthcare in patients with T2D.

**Trial registration:**

Nederlands Trialregister (NTR6166), 6 Jan 2017.

## Background

Patients diagnosed with type 2 diabetes (T2D) are at increased risk for developing cancer; especially the risk of obesity-related cancers [1–5]. According to the most recent World Cancer Research Fund (WRCF) definitions obesity related cancers include oesophageal cancer, liver cancer, kidney cancer, stomach cardia cancer, colorectal cancer, advanced prostate cancer, post-menopausal breast cancer, gallbladder cancer, pancreatic cancer, ovarian cancer and endometrial cancer [6].

In addition to T2D and obesity, glucose-lowering agents used in the treatment of T2D have also been associated with cancer risk and some studies have reported that these relations can be drug specific. For example, the use of pioglitazone, not rosiglitazone, has been linked to the development of bladder cancer in some studies [7, 8], although the robustness of the evidence underlying this possible relationship remains unclear and was absent in recent reports [9, 10]. Also, insulin glargine has been linked to higher breast cancer risk in some studies [11], although - again - several studies reported no or even an inverse association [11–13]. Metformin has more consistently been associated with a decreased cancer risk [14], however concerns have been raised that this association might have been influenced by several types of bias [15, 16].

The sulfonylureas (SUs) are one out of six classes of oral glucose-lowering agents advised by the EASD and ADA as a second step when the glycaemic treatment targets are not reached with metformin mono-therapy [17]. Sulfonylureas have been available for many years and are highly efficacious at low costs. In the Dutch primary care treatment guideline for T2D, gliclazide is the preferred SU, as opposed to both the ADA and EASD which do not recommend a specific SU [17]. Previous studies have shown that within the class of SUs differences exist with regard to hypoglycaemia risk [18], for example there have been no reports of severe hypoglycaemia events in gliclazide users [18]. In addition, within-class differences in risk of cardiovascular events and safety when prescribed to patients with renal failure have been reported [19, 20].

An association between the class of SUs and increased overall cancer risk has also been reported [21–24]. Most previous studies are limited by methodological issues, for example many studies reported baseline SU use and did not account for duration of SU use [22–24]. There is also evidence suggesting within-class SU differences in cancer risk, where gliclazide use has been associated with a lower cancer risk [21–24]. There are several potential mechanistic explanations, one of which could be that gliclazide leads to a more selective glucose dependent insulin response and lower insulin levels. In what way and to what degree cancer risk is modified by different SUs in unclear and requires further investigation and confirmation.

Most evidence, however, is derived from small observational cohort studies and substantial knowledge gaps exist. This also holds true for the presumed favourable long-term cancer safety profile of gliclazide in particular. The relations between use of glucose lowering agents and cancer are complex and there is overlap in risk factors; for example, several glucose lowering agents have been associated with weight gain, which in itself has also been related to an increased cancer risk.

The primary aim of this study is to evaluate within-class SU differences in risk for obesity-related cancer (excluding non-melanoma skin cancers) accounting for weight changes during follow-up and drug exposure [6]. Secondary aims are to evaluate within-class SU differences concerning all cancers combined and the cancer risks of the five largest groups of site specific cancers (breast, colorectal, prostate, bladder and lung cancer) accounting for duration of drug use.

## Methods/design

### Data source

This study will be conducted using a combined database of the ZODIAC (Zwolle Outpatient Diabetes project Integrating Available Care) study and NCR (Dutch National Cancer Registration).

#### ZODIAC

The ZODIAC cohort is part of an ongoing primary care prospective study initiated in 1998, in which annually collected data are used for care improvement, benchmarking and research [25]. Patients consented with the anonymous use of their data for study purposes. Patients included are diagnosed with T2D and are exclusively treated in primary care in a shared care setting. Data on age, sex, date of T2D diagnosis, HbA1c, length, weight, estimated GFR, creatinine, albumin-creatinine ratio (ACR), cholesterol/HDL ratio, blood pressure, macrovascular complications (myocardial infarction, TIA, CVA) medication use (both diabetes-specific and other medication), smoking (yes/no) and alcohol use (yes/no) are recorded.

#### NCR

The Netherlands Cancer Registration (NCR) was founded in 1989 and has since recorded almost every cancer event in the Netherlands, and includes incidence date, TNM (tumour, node, metastasis) stage, morphology, location and the therapy received [26]. Basal cell carcinoma of the skin, carcinoma in situ of the cervix, myelodysplastic syndrome and myeloproliferative disorders are all excluded for the NCR database. Benign and borderline tumours are excluded with the following exceptions; benign brain tumours (included from 1999), carcinoids of the appendix (included from 2001), borderline tumours of the ovaries (included from 2001), thymoma (included from 2001), phyllodes tumours (included from 2001) and T-cell leukaemia (included from 2004).

### Study population

#### Combined ZODIAC-NCR cohort

All cancer events that occurred between 1 January 1989 and 2012 were linked to the data of the ZODIAC study via a trusted third party using postal code, full name, date of birth and sex. The medical ethics committee of Isala, Zwolle, the Netherlands approved the linkage of ZODIAC and NCR (METC reference number 13.0765). The NCR expected that the number of false-positive and the number false-negative linkage is both under 1%. By combining the two databases a cohort composed of patients diagnosed with T2D between 1 January 1998 and 31 December 2012 was assembled.

#### Patient selection

The study cohort entry date and baseline date will be the date the patients started participation in the ZODIAC cohort.

#### Inclusion

All patients included will be participating in the ZODIAC-NCR cohort on or after January 1998 and will be users of SUs.

#### Exclusion

Patients treated with long-acting or mixed insulin before oral glucose lowering agents and those receiving insulin on top of SU at study entry will be excluded. Patients who received a cancer diagnosis before receiving a SU will be excluded. For the main analyses patients who switch medication within the class of SUs will be excluded at the time the switch occurs.

#### Follow-up

All patients will be followed from the year of cohort entry until a diagnosis of cancer [3–5]. Patients with no diagnosis of cancer will be censored at the time of death, end of registration within the ZODIAC cohort or end of the study period (31 December 2012), whichever occurred first.

#### Study endpoints

The primary outcome will be within-class SU difference in obesity-related cancer risk (see Table 1 for included cancers). The secondary outcomes will be all cancer risk (Table 2), site-specific cancer risk and the presence of a duration response relationship between SU use and cancer. Cancer sites of special interest will be specific cancers of the breast, colorectal, bladder, advanced prostate and lung cancer. Study endpoints will be evaluated for men and women separately.

**Table 1:** Cancers included in primary endpoint obesity related cancer

Men	Women
Oesophageal (adenocarcinoma)	Oesophageal (adenocarcinoma)
Stomach cardia	Stomach cardia
Kidney	Kidney
Gallbladder	Gallbladder
Liver	Liver
Pancreatic	Pancreatic
Colorectal	Colorectal
Prostate cancer	Ovarian
	Breast
	Endometrial

**Table 2:** Cancer excluded from secondary endpoint all cancer risk

Men (cancers excluded)	Women (cancers excluded)
Non-melanoma skin cancer	Non-melanoma skin cancer
Breast	Prostate
Ovarian	Male genital organs
Endometrial	
Female genital organs	

#### Exposure

Patients will be considered unexposed to SUs until the time of the first SU prescription within ZODIAC. A one-year lag period will be accounted for. A lag period is necessary to take into account a latency time window and to minimise possible detection bias around the time of treatment initiation. Exposure to a SU will be classified according to one of the following, mutually exclusive, categories: gliclazide use, glimepiride use, tolbutamide use, glibenclamide use, non-SU use.

We aim to determine whether there are duration-response relationships between the use of SUs and obesity-related cancer incidence. Duration-response will be assessed in terms of cumulative duration of use, defined as the total number of years of use calculated by summing the durations of yearly prescriptions received between cohort entry and the time of the event and will be used as a time-dependent covariate.

#### Co-variates

Co-variates collected at cohort entry and annually thereafter are: age, sex, year of cohort entry, HbA1c (continuous), diabetes duration (time between diabetes diagnosis and cohort entry, continuous), BMI (continuous), serum creatinine (continuous), metformin use (yes, no), insulin use (yes/no), history of cancer (no non-melanoma skin cancer) (yes, no) and smoking (ever, never, unknown).

#### Primary analysis

Descriptive statistics will be used to characterize the patients at cohort entry.

Time dependent cox proportional hazard analyses will be used to estimate the adjusted hazard ratio of developing obesity-related cancer when using gliclazide compared to other SUs (both individual and grouped as non-gliclazide SU). Exposure to SUs will be included as the cumulative number of years exposed to a specific SUs. Exposure status for SU will be updated annually. The primary analyses will be corrected for the previously mentioned confounders measured at baseline.

#### Secondary analyses

In a secondary analyses changes diabetes medication during follow-up will be accounted for. Time dependent cox proportional hazard analyses will be used to estimate the adjusted hazard ratio of developing all cancer when using gliclazide compared to other SUs (both individual and grouped as non-gliclazide SU). Exposure to confounders (including concurrent metformin and insulin use) will be handled as time varying variables where follow-up is available. The updated mean method will be used for HbA1c, BMI and serum creatinine. These analyses will be repeated to investigate the adjusted hazard ratio of breast, colorectal, prostate, bladder and lung cancer.

#### Missing data

When appropriate, in case of missing data multiple imputation will be used. In case multiple imputation cannot be used (e.g. data are not missing at random or missing completely at random), the updated means method will be used. The updated mean method averages the baseline values with the mean annual values [27]. The updated mean method is similar to the technique used in the UKPDS [28]. When calculating the updated means, we will allow a maximum of 2 consecutive years to be missing, with a maximum of 3 years in the complete follow-up.

#### Subgroup analyses

In subgroup analyses effects of exposure to BMI and HbA1c during follow-up and the relation between SUs and cancer will be investigated and interaction will be tested. A second subgroup analysis will investigate cancer risk in patients who do and do not use metformin in combination with an SU.

#### Sensitivity analysis

Six sensitivity analyses will be planned for supporting the main analyses. Firstly, because the latency window is uncertain, the primary analyses for within-class differences will be repeated with lag periods of zero and two years. Secondly, the primary analysis will be repeated but the adjusting confounders will be measured at the year before first SU prescription. Thirdly, the main analysis will be repeated but with the exclusion of cancer events 1 year after initiation of a SU. Fourthly, to investigate the accuracy of our results the analysis will be repeated in patients in whom all data on medication are complete. Fifthly, to investigate the accuracy of our results the analysis will be repeated in patients who have no missing data on HbA1c, BMI and serum creatinine. Sixthly, to quantify the effect of patients switching medication within the class of SUs, an intention-to-treat analysis for patients who switch SUs will be performed.

## Discussion

This study will be the first large observational cohort study investigating differences in cancer risk within the class of SUs. An estimated 8% of the global population is known with T2D [29] and this could translate into an increased risk of cancer for an substantial amount of people. The prevalence of T2DM is expected to rise evermore, at least for the next decades [30]. A minimal change in cancer risk could result in a substantial change in the relative and even absolute number of patients diagnosed with cancer. If this study confirms the presence of within-class SU cancer risk differences, it could help patients and physicians in making a shared decision for a specific SU. This could contribute to quality of life of the patients as well as contribute to increasing effective care and cost-effectiveness of healthcare. If no differences are present, the safety, efficacy, and cost of SU will remain the only criteria for selecting the best SU.
